# Ketamine inhibits TNF-α-induced cecal damage by enhancing RIP1 ubiquitination to attenuate lethal SIRS

**DOI:** 10.1038/s41420-022-00869-x

**Published:** 2022-02-19

**Authors:** Bin Deng, Daowei Yang, Huanghui Wu, Lu Wang, Rui Wu, Hongrui Zhu, Ailing Huang, Jingyi Song, Tieliang Cai, Shanshan Liu, Jingsi Wu, Huiying Zhou, Chunhui Li

**Affiliations:** 1 Department of Anesthesiology, Xiang’an Hospital of Xiamen University, School of Medicine, Xiamen University. Xiamen, Xiamen, 361101 China; 2 Department of Anesthesiology, The First Affiliated Hospital of Xi’an Jiaotong University, Xi’an, 710061 China; 3 Department of Clinical Sciences, , Malmö, Lund University, Jan Waldenströms Gata 35, Malmö, 214 28 Sweden; 4 Department of Anesthesiology, Affiliated Hospital of Jiaxing University, The First Hospital of Jiaxing, Jiaxing, Zhejiang 314000 China; 5 State Key Laboratory of Cellular Stress Biology, Xiamen University. Xiamen, Xiamen, 361101 China; 6 Department of Anesthesiology, Chenggong Hospital of Xiamen University, Xiamen, 361000 China

**Keywords:** Necroptosis, Mechanisms of disease, Inflammatory diseases

## Abstract

Systemic inflammatory response syndrome (SIRS) is a sepsis-associated inflammatory state and a self-defense mechanism against specific and nonspecific stimuli. Ketamine influences many key processes that are altered during sepsis. However, the underlying mechanisms remain incompletely understood. In this study, TNF-α-treated mice, as well as HT-29 and L929 cell models, were applied to characterize TNF-α-induced systemic and local cecal tissue inflammatory responses. Behavioral, biochemical, histological, and molecular biological approaches were applied to illustrate the related processes. Mice with TNF-α-induced SIRS showed systemic and local cecal tissue inflammatory responses, as indicated by increased levels of high mobility group box 1 protein (HMGB1), chemokines (C-X-C motif) ligand 10 (CXCL10), interleukin-6 (IL-6), and IL-10, as well as high mortality. Ketamine pretreatment alleviated death rates, symptoms, and the production of inflammatory cytokines induced by TNF-α in mice. Moreover, ketamine also protected the mice from TNF-α-induced cecal damage by suppressing the phosphorylation of receptor-interacting serine/threonine-protein kinase 3 (RIP3) and mixed lineage kinase domain-like protein (MLKL). In addition, our results showed that ketamine efficiently inhibited TNF-α-induced necroptosis in HT-29 and L929 cells. Furthermore, we explored the mechanism using different L929 cell lines. The results displayed that ketamine inhibited TNF-α-induced necroptosis by enhancing RIP1 ubiquitination and reducing the RIP1-RIP3 and RIP3-MLKL interactions, as well as the formation of necrosomes. Thus, our study may provide a new theoretical and experimental basis for treating diseases characterized by SIRS-associated inflammatory factor storms. Moreover, our exploration may provide potential molecular mechanisms and targets for therapeutic intervention and clinical application of ketamine.

## Introduction

Systemic inflammatory response syndrome (SIRS) is characterized by innate immune system activation and the stimulation of inflammatory responses, followed by a cytokine storm in the circulation, which involves the generation and secretion of proinflammatory cytokines [1]. As a consequence, the uncontrolled inflammatory response causes sepsis to be a life-threatening organ dysfunction [2]. In the early and late stages of sepsis, immune cell activation triggers a complicated signal transduction pathway resulting in activating nuclear factor-kappa B (NF-κB) and mitogen-activated protein kinase (MAPK), followed by releasing a large number of cytokines and chemokines and complement system activation [3–6]. Previous studies suggested that lipopolysaccharide (LPS)-induced macrophages primarily release inflammatory mediators including interleukin-6 (IL-6) and tumor necrosis factor-alpha (TNF-α), and secondary mediators taking part in pathogen removal, including reactive oxygen species (ROS) [7, 8]. To date, the pathogenesis of SIRS remains unclear. To understand the mechanism, animal models have been developed, and many investigators have used TNF-α-induced SIRS mouse models of sterile sepsis [9, 10].

It shall be noted that cecum was illustrated as especially sensitive to TNF-α-induced damage. Previous researches have strongly shown that TNF-α could trigger cecal epithelial necroptosis, a kind of programmed cell death associated with the proteins receptor-interacting protein kinase 1 (RIP1), RIP3, and mixed lineage kinase domain-like protein (MLKL) [11–13]. More importantly, pharmaceutical inhibition cecal epithelial necroptosis strikingly promoted the survival in mice receiving TNF-α [13], indicating the preliminary role of cecal epithelial necroptosis in TNF-α-induced cecal damage and SIRS. According to previous studies, TNF receptor 1 (TNFR1) trimerizes with RIP1 and TNFR1-associated death domain (DD) protein (TRADD) after TNF-α stimulation, resulting in the formation of membrane-associated protein complex I [14]. In addition, RIP1 activation may alternatively result in life-or-death decisions in the cell, relying on the cell type and context [15]. RIP1 ubiquitination is required for activation of the IκB kinase (IKK) complex [16], IκB-α degradation, and NF-κB activation. However, RIP1 deubiquitination disables its prosurvival function and leads to apoptosis or necroptosis [17]. When caspase-8 is absent or its activity is blocked, activated RIP1 can interact with RIP3 through the RHIM-RHIM domain, causing polymerized and phosphorylated RIP3 to form complex IIb, which is composed of RIP1/RIP3 [18]. Ultimately, RIP3-induced MLKL phosphorylation induces necroptosis by disrupting plasma membrane integrity [19–21]. Necrostatins were the first RIP1 inhibitors identified and affect the necroptosis pathway [22], but it has been disclosed that no drugs, such as necrostatins exert preclinical effects. Herein, ketamine was recognized as a strong inhibitor of necroptosis in vitro and in vivo.

Ketamine has been widely administered in clinical anesthesia as a noncompetitive glutamatergic N-methyl-D-aspartate receptor (NMDAR) antagonist [23]. According to current research, ketamine plays rapid antidepressant [24], anti-inflammatory, and immunomodulatory roles [25, 26]. Ketamine has been applied to sepsis [27] in macrophages stimulated with endotoxin, NF-κB, and activator protein-1 (AP-1) and affects neutrophil function and the production of inflammatory molecules such as TNF-α and interleukins [23, 26, 28]. However, the role of ketamine on TNF-α-induced cecal damage and epithelial necroptosis is far from being revealed.

The current research demonstrated that ketamine could ameliorate TNF-α-induced death in C57BL/6 mice as well as HT-29 and L929 cells. Furthermore, we showed that ketamine suppressed the phosphorylation of RIP3 and MLKL by enhancing the ubiquitination levels of RIP1. Thus, we concluded that ketamine could protect mice from TNF-α-induced death and will be a potent therapeutic drug for SIRS.

## Results

### Ketamine alleviated death and sickness responses induced by TNF-α in mice

**Fig. 1:**
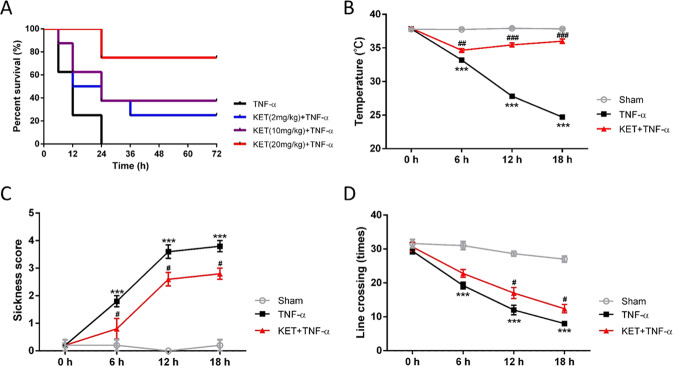
Ketamine alleviated the death and sickness response induced by TNF-α in mice. **A** Effects of different ketamine concentrations on the survival of model animals stimulated with TNF-α. **B**–**D** The effects of ketamine (20 mg/kg) on the temperatures, sickness behaviors, and sickness scores of mice after TNF-α-induced SIRS. All data are shown as the mean ± SEM. *n* = 7/group. *Significantly different from the sham group; ^#^significantly different from the TNF-α group. ***p < 0.001, ^#^*p* < 0.05, ^##^*p* < 0.01, ^###^*p* < 0.001.

It has been reported that TNF-α can induce death within 24 h in mice. In this study, 20 min after prophylactic ketamine was injected intraperitoneally, TNF-α was injected via the tail vein into the mice. The outcomes displayed that after TNF-α (15 µg) injection, all mice died within 24 h. In contrast, prophylactic ketamine greatly boosted the survival of mice in a dose-dependent manner. In particular, the percent survival of mice in the KET + TNF-α group was 71.4% (Fig. 1A). Besides, we also investigated the protective effect of therapeutic ketamine (2, 10, and 20 mg/kg 20 min after TNF-α injection), as well as prophylactic (1, 5, and 10 mg/kg 20 min before TNF-α injection) combined with therapeutic ketamine (1, 5, and 10 mg/kg 20 min after TNF-α injection) on survival rate in mice with SIRS. Notably, the protective effect of therapeutic ketamine was limited during 72 h, even though receiving a high dose of ketamine (2 mg/kg *vs*. 10 mg/kg *vs*. 20 mg/kg: 0% *vs*. 10% *vs*. 30%. Supplementary Fig. 1A). Interestingly, the survival rate in mice receiving prophylactic combined with therapeutic ketamine was similar with prophylactic ketamine only (prophylactic combined with therapeutic ketamine *vs*. prophylactic ketamine: 70% vs. 71.4%. Supplementary Fig. 1B). Therefore, the prophylactic mechanism was further investigated in the following work. By comparing with those in the TNF-α group, the temperatures of mice in the KET (20 mg/kg)+TNF-α group grew significantly at 6 h (*p* < 0.001), 12 h (*p* < 0.001) and 18 h (*p* < 0.001) after TNF-α administration (Fig. 1B). Furthermore, sickness behavior was significantly influenced by TNF-α and ketamine. By comparing with those in the TNF-α group, the sickness scores of mice in the KET (20 mg/kg) + TNF-α group were increased at 6 h (*p* < 0.05), 12 h (*p* < 0.05) and 18 h (*p* < 0.05) after TNF-α administration (Fig. 1C). Line crossing was also used to test sickness behavior. By comparing with those in the TNF-α group, the numbers of line crossings of mice in the KET (20 mg/kg) +TNF-α group grew at 12 h (*p* < 0.05) and 18 h (*p* < 0.05) after TNF-α administration (Fig. 1D).

### Ketamine decreased the expression of inflammatory cytokines in mouse serum

**Fig. 2:**
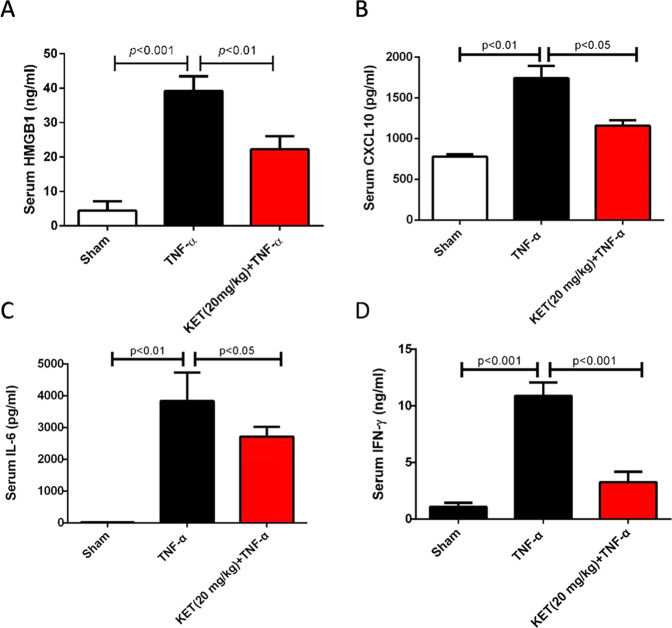
Ketamine decreased the expression of inflammatory cytokines in mouse serum. **A** Serum HMGB1 from mice with indicated treatment was measured at 12 h after TNF-α administration by HMGB1 ELISA kit. **B** Serum CXCL10 from mice with indicated treatment was measured at 12 h after TNF-α administration by CXCL10 ELISA kit. **C** Serum IL-6 from mice with indicated treatment was measured at 12 h after TNF-α administration by IL-6 ELISA kit. **D** Serum IFN-γ from mice with indicated treatment was measured at 12 h after TNF-α administration by IFN-γ ELISA kit. All data are shown as the mean ± SEM. *n* = 7/group.

At 12 h after TNF-α administration, we measured the levels of HMGB1, IL-6, CXCL10, and IFN-γ in the serum of the mice. The results showed that compared with those in the sham group, the levels of HMGB1, IL-6, CXCL10, and IFN-γ in the TNF-α group increased significantly at 12 h after TNF-α administration. By comparing with those in the TNF-α group, the levels of HMGB1, IL-6, CXCL10, and IFN-γ decreased in the KET (20 mg/kg) + TNF-α group (Fig. 2A–D).

### Ketamine protected mice from TNF-α-induced cecal damage by suppressing the phosphorylation of RIP3 and MLKL

**Fig. 3:**
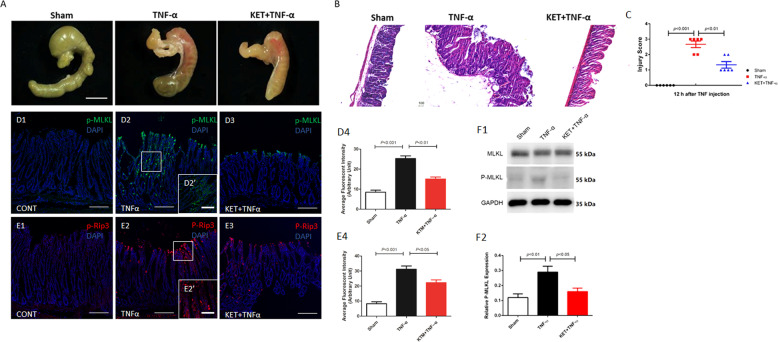
Ketamine protected mice from TNF**-α**-induced cecal damage by suppressing the phosphorylation of RIP3 and MLKL. **A** Representative images of cecal tissue from mice in each group at 12 h after the injection of 15 μg of TNF-α. **B** When a mouse became moribund, tissues were collected, sectioned, and stained with H&E. Representative images are shown. Scale bar, 100 μm. TNF-induced cecal damage at the indicated time was scored and is shown in **C**. **D**, **E** Immunofluorescent staining and statistical analysis of the expression of the necroptosis markers p-MLKL and p-RIP3 in cecal tissue from mice in each group at 12 h after the injection pf 15 μg of TNF-α. **F** Western blot analysis of the expression of the necroptosis markers MLKL, p-MLKL in cecal tissue from mice in each group at 12 h after the injection pf 15 μg of TNF-α. All data are shown as the mean ± SEM. *n* = 4/group.

It is worth noting that the cecum is sensitive to TNF-α-induced damage in the TNF-SIRS model, which has been reported in previous studies [29, 30]. Our results showed that the cecal tissue was hyperemic and edematous in mice in the TNF-α group at 12 h after TNF-α administration, as observed by stereomicroscopy. Interestingly, cecal damage was attenuated by ketamine pretreatment (Fig. 3A). H&E staining showed inflammatory cell infiltration, hyperemia, and epithelial cell damage in tissue sections from the TNF-α group, and these effects were alleviated by ketamine pretreatment (Fig. 3B). By comparing with that in the sham group, the damage score of cecal tissue in the TNF-α group increased significantly at 12 h after TNF-α administration (*p* < 0.001). By comparing with that in the TNF-α group, the damage score of cecal tissue decreased in the KET + TNF-α group (*p* < 0.001) (Fig. 3C). It has been reported that TNF**-**α-induced necroptosis is essential for TNF-induced SIRS, because RIP3***-***KO mice resist TNF-induced death [31, 32]. Among the spleen, small intestine, thymus, liver, kidney, lung, heart, pancreas, and cecum, the cecum was the only organ in which damage was attenuated by RIP3-KO [30]. Our results showed that the TNF-α group showed higher expression of p-RIP3 and p-MLKL in cecal tissue than the sham group at 12 h after TNF-α administration. Compared with that in the TNF-α group, the expression of p-RIP3 and p-MLKL decreased in the KET + TNF-α group in immunofluorescent staining. Furthermore, by comparing with that in the TNF-α group, the expression of p-MLKL declined in the KET + TNF-α group in western blot (Fig. 3D–F).

### Ketamine improved the survival of HT-29 cells and suppressed the level of MLKL phosphorylation

**Fig. 4:**
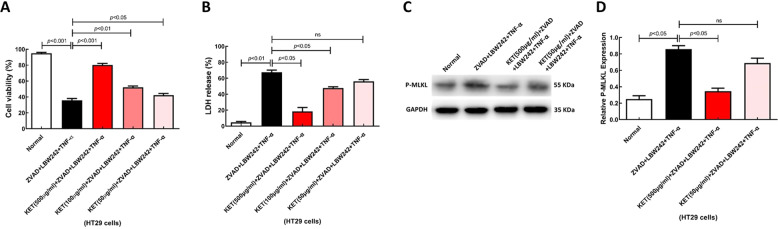
The effect of ketamine on ZVAD/LBW242/TNF-α-induced the survival of HT-29 cells and suppressed the level of MLKL phosphorylation. HT-29 cells were treated with 10 µM Smac mimetic LBW242 and 20 µM ZVAD for 1 hour prior to stimulation with 20 ng/ml rhTNF in combination with different concentrations of ketamine for 24 h. **A** Cell viability via measuring ATP level in every group. **B** LDH release was used to determine the survival of HT-29 cells. **C**, **D** Immunoblot analysis of p-MLKL and GAPDH in HT-29 cells. Quantitative data are shown as the mean values of three independent experiments and the error bars are the mean ± SEM, ns no significant difference.

Our outcomes displayed that ZVAD/LBW242/TNF-α management greatly decreased cell viability, and ketamine obviously decreased ZVAD/LBW242/TNF-α-induced cell damage and inhibit LDH release in HT-29 cells in a dose-dependent way (Fig. 4A, B). Meanwhile, ketamine (500 μg/ml) significantly suppressed the phosphorylation of MLKL in HT-29 cells after ZVAD/LBW242/TNF-α (Fig. 4C, D). Thus, these data suggested that ketamine could suppress suppressed-induced necroptosis in HT-29 cells.

### Ketamine effectively alleviated TNF-induced damage and ROS accumulation in L929 cells

**Fig. 5:**
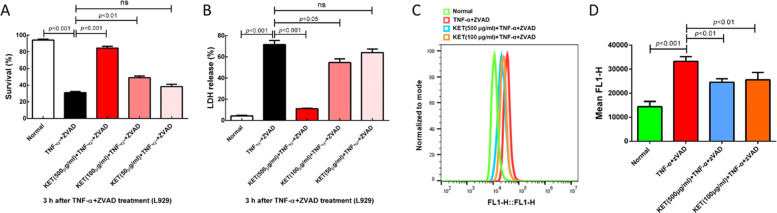
The effect of ketamine on TNF-induced damage and ROS accumulation in L929 cells. L929 cells were treated with nothing (Normal group), TNF-α + ZVAD, or TNF-α + ZVAD plus different doses of ketamine as indicated for 3 h. **A** The survival rates of cells were determined by PI exclusion using flow cytometry. **B** Lactate dehydrogenase (LDH) release was used to determine the number of dead cells. **C**, **D** Representative ROS production was analyzed by quantitative analysis by flow cytometry. ns no significant difference.

Next, we wanted to clarify the mechanism by which ketamine protects against the TNF-α-induced necroptosis pathway. Because TNF-α can trigger necroptosis in L929 cells, we examined the role of ketamine in the inhibition of TNF-induced necroptosis in L929 cells. The experiment included ZVAD to exclude apoptosis. The results showed that ketamine could efficiently promote cell survival and inhibit LDH release in a dose-dependent manner at 3 h after TNF-α and ZVAD administration (Fig. 5A, B). Studies have indicated that mitochondrial ROS take part in some necroptosis but not all types of cells [33]. Our in vitro flow cytometry outcomes revealed that ROS levels were greatly upregulated in the TNF-α + ZVAD group compared with the normal group at 3 h after TNF-α plus ZVAD administration. In addition, the flow cytometry outcomes showed the downregulation of ROS levels after ketamine administration by comparing with those in the TNF-α + ZVAD group (Fig. 5C, D).

### Ketamine efficiently inhibited TNF-α-induced necroptosis in L929 cells

**Fig. 6:**
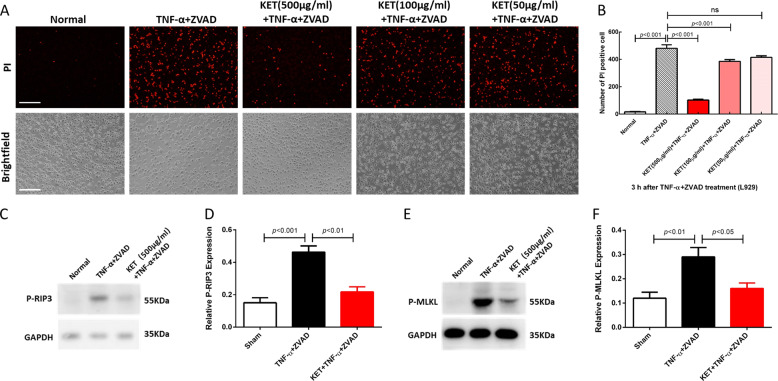
The effect of ketamine on TNF-induced necroptosis in L929 cells. L929 cells were treated with nothing (Normal group), TNF-α + ZVAD, or TNF-α + ZVAD plus different doses of ketamine as indicated for 3 h. **A**, **B** The cells were stained with PI and analyzed by immunofluorescence staining and bright field microscopy. PI-positive cells represent dead cells and were counted in three independent experiments. **C**–**F** Immunoblot analysis of p-RIP3, p-MLKL, and GAPDH in L929 cells treated with nothing (Normal group), TNF-α + ZVAD, or TNF-α + ZVAD plus ketamine for 3 h. Quantitative data are shown as the mean values of three independent experiments and the error bars are the mean ± SEM., ns no significant difference.

The results indicated that the TNF-α + ZVAD group showed more PI-positive cells than the normal group at 3 h after TNF-α and ZVAD administration. By comparison, the KET (500 μg/ml) + TNF-α + ZVAD group and KET (100 μg/ml) +l TNF-α + ZVAD group showed less PI-positive cells than the TNF-α + ZVAD group, indicating that ketamine prevented necroptosis (Fig. 6A, B). As anticipated, ketamine significantly blocked the phosphorylation of RIP3 and MLKL in L929 cells after TNF-α and ZVAD stimulation (Fig. 6C–F). Thus, these data suggested that ketamine could suppress TNF-α- and ZVAD-induced necroptosis in L929 cells.

### Ketamine targeted RIP1 or factors upstream of RIP1 in TNF-α-induced L929 cell necroptosis

**Fig. 7:**
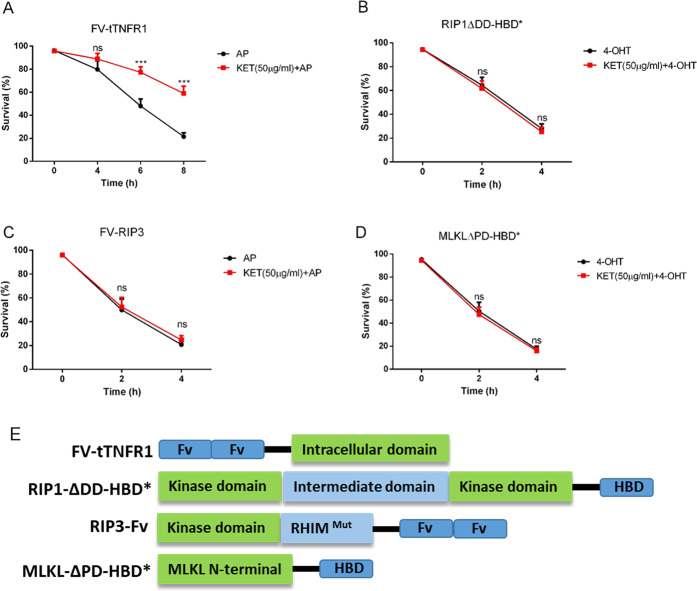
Ketamine targeted RIP1 or factors upstream of RIP1 in TNF-αTNF-α-induced L929 cell necroptosis. **A**–**D** Lentivirus expressing proteins described in **E** was used to infect corresponding KO L929 cells. These cells were treated with 4-OHT for the HBD*-fused protein and AP20187 for the FV-fused protein at the indicated times with or without ketamine. Cell death was analyzed by flow cytometry, and the quantitative data are the mean values of three independent experiments. The error bars are the mean ± SEM., ***p < 0.001, ns: no significant difference. **E** Schematic illustration of FV-fused tTNFR1, RIP3, and HBD*-fused RIP1ΔDD; MLKLΔPD; HBD* represents the HBD-G521R mutation; RHIM^mut^ represents the QIG449-451AAA mutation.

Because TNF-α-induced necroptosis in L929 cells is mediated through the TNFR1-RIP1-RIP3-MLKL signaling pathway, we examined whether the target of ketamine was downstream of TNFR1, RIP1, RIP3, and MLKL. It has been shown that the dimerization or oligomerization of TNFR1, RIP1, RIP3, or MLKL can dramatically induce necroptosis. Therefore, we used this system to clarify the role of ketamine in necroptosis. Artificial dimerization systems were on basis of the FK506 binding domain F36V mutant (FV) or hormone-binding domain G521R mutant (HBD*). We stably transfected FV-tTNFR1, RIP1ΔDD-HBD*, FV-RIP3, and MLKLΔPD-HBD* into the corresponding KO L929 cells. Upon treatment of L929 cells expressing RIP1ΔDD-HBD* or MLKLΔPD-HBD* with 4-OHT and L929 cells expressing FV-tTNFR1 or FV-RIP3 with AP20187, abundant cell death was found within several hours. According to Fig. 7A, ketamine efficiently inhibited cell death induced by FV-tTNFR1 oligomerization. In contrast, cell death induced by the oligomerization of RIP1ΔDD-HBD*-, FV-RIP3-, and MLKLΔPD-HBD* was not blocked by ketamine (Fig. 7B–D). Collectively, these data demonstrated that ketamine acted on or upstream of RIP1 in the TNF-α-induced necroptosis pathway.

### Ketamine enhanced RIP1 ubiquitination after TNF-α stimulation

**Fig. 8:**
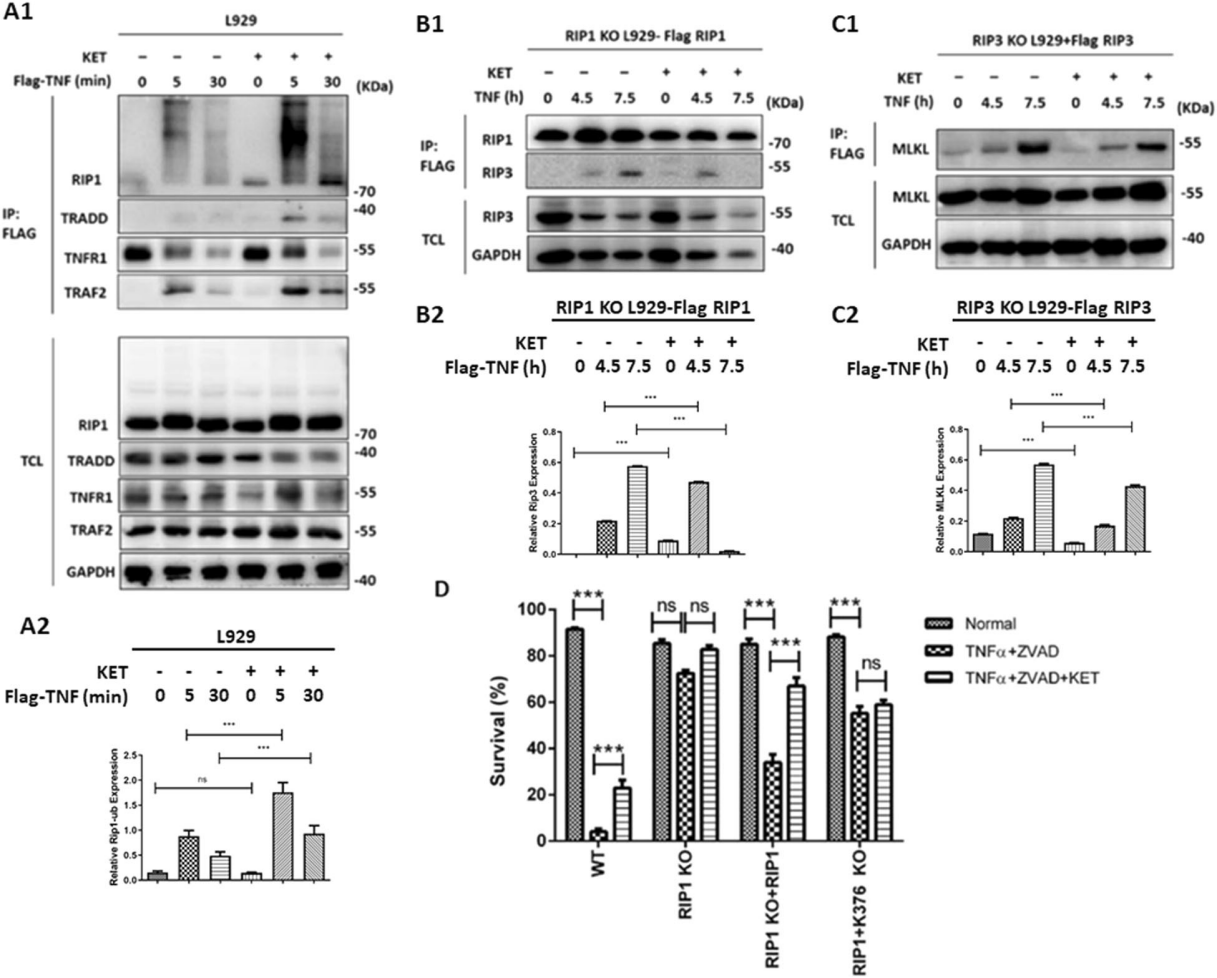
Ketamine enhanced RIP1 ubiquitination after TNF-α stimulation. **A** L929 cells were treated with 3 × Flag-TNF-α (100 ng/ml) with or without ketamine (500 µg/ml) for the indicated time. Cell lysates were immunoprecipitated with mouse anti-Flag M2 beads and analyzed by western blotting with the indicated antibodies. **B**, **C** Flag-RIP1-expressing RIP1-KO L929 cells (**B**) or Flag-RIP3-expressing RIP3-KO L929 cells (**C**) were treated with TNF-α (10 ng/ml) or TNF-α (10 ng/ml) plus ketamine (500 µg/ml) for the indicated time. Cell lysates were immunoprecipitated with mouse anti-Flag M2 beads and analyzed by western blotting with the indicated antibodies. **D** WT, RIP1-KO, and WT RIP1- or K376R RIP1-expressing RIP1 L929 cells were treated with nothing (Normal), TNF-α (10 ng/ml) plus ZVAD (20 µM) or TNF-α (10 ng/ml) plus ZVAD (20 µM) and ketamine (500 µg/ml) for 2.5 h. Cells were stained with PI, and cell survival was analyzed by flow cytometry. The data are the mean values of three independent experiments, and the error bars are the mean ± SEM., ***p < 0.001, ns no significant difference.

After TNF binds to the receptor, TNFR1 will recruit multiple proteins to form the complex I. Polyubiquitination of RIP1 in complex I mediated by cIAP ligases is very important for the activation of NF-κB and mitogen-activated protein kinases (MAPKs). Inhibition of the ubiquitination of RIP1 will enhance cell death. Ketamine treatment delayed TNFR1 oligomerization- but not RIP1/RIP3/MLKL oligomerization-induced necroptosis, suggesting that ketamine treatment might affect the formation of complex I. Complex I was analyzed by Flag-TNF-α immunoprecipitation with or without ketamine. As shown in Fig. 8A, the ubiquitination of RIP1 in complex I was obviously enhanced with ketamine treatment. The RIP1-RIP3 and RIP3-MLKL interactions were also analyzed by Flag-RIP1 or Flag-RIP3 immunoprecipitation in RIP1-KO L929 cells showing Flag-RIP1 and RIP3-KO L929 cells showing Flag-RIP3 after TNF-α stimulation with or without ketamine. As shown in Fig. 8B, C, after ketamine treatment, the interactions between RIP1-RIP3 and RIP3-MLKL induced by TNF-α were significantly decreased.

RIP1 ubiquitination has been reported to play an essential role in activating NF-κB phosphorylation and inhibiting the formation of necrosomes. NF-κB activation reduced TNF-α-induced necroptosis through the upregulation of some anti-death proteins, such as c-FLIP, while ketamine treatment enhanced the degradation of IκB-α and increased NF-κB activation (Supplementary Fig. 2A). However, ketamine treatment still stopped TNF-α-induced necroptosis in NEMO-KO L929 cells, and the NF-κB signal was abolished (Supplementary Fig. 2B), suggesting that NF-κB activation is unnecessary for ketamine-mediated inhibition of necroptosis.

After TNF-α stimulation, RIP1 is ubiquitinated at different sites in different ways, and lysine 376 has been reported to be the most important site. TNF-α-induced NF-κB activation was decreased and TNF-α-induced cell death was significantly increased in K376R RIP1 mutant cells. To determine whether RIP1 ubiquitination is essential for ketamine-mediated inhibition of necroptosis, we rescued wild-type (WT) RIP1 and K376R RIP1 in RIP1-KO L929 cells and treated these cells without TNF-α or with ketamine. To exclude apoptosis induced by changes in RIP1 protein levels, ZVAD was added in combination with TNF-α (Fig. 8D). Ketamine treatment only inhibited necroptosis induced by TNF-α and ZVAD in RIP1- but not RIP1-K376R-expressing L929 cells, suggesting that RIP1 ubiquitination is necessary for ketamine-mediated inhibition of TNF-α-induced necroptosis. Furthermore, we also confirmed the RIP1 ubiquitination in vivo. Our finding suggested a significantly increased of RIP1 ubiquitination expression in cecal tissue in SIRS mice pre-treated with ketamine (Supplementary Fig. 3A, B). These results suggest that ketamine treatment could increase the ubiquitination of RIP1, reducing the RIP1-RIP3 and RIP3-MLKL interactions, as well as the formation of necrosomes, and resulting in the inhibition of TNF-α-induced necroptosis. However, the mechanism by which RIP1 ubiquitination is increased by ketamine requires further investigation.

## Discussion

**Fig. 9:**
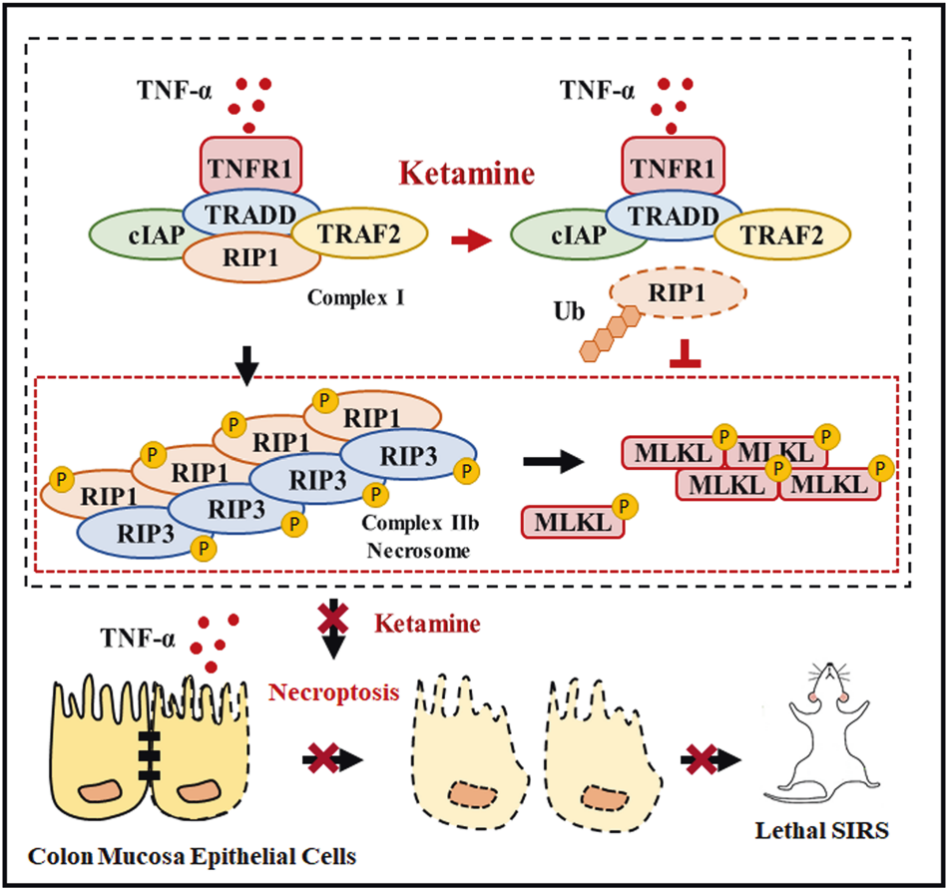
Schematic diagram summarizing the proposed mechanism by which ketamine alleviates the lethal systemic inflammatory response by inhibiting cecal damage through promoting RIP1 ubiquitination. The pathogenesis of TNF-α-induced SIRS is associated with cecal damage via RIP1-, RIP3-, and MLKL-related signaling pathways. Interestingly, we found that ketamine efficiently inhibited TNF-α-induced cecal damage by enhancing RIP1 ubiquitination and reducing RIP1-RIP3 and RIP3-MLKL interactions, as well as the formation of necrosomes, which was beneficial for attenuating lethal SIRS.

The current research showed that ketamine alleviated the death and sickness response induced by TNF-α in mice. Then, we demonstrated that ketamine attenuated TNF-α-induced cecal damage by suppressing the phosphorylation of RIP3 and MLKL. Furthermore, this study showed that ketamine reduced the TNF-α-induced serum levels of inflammatory cytokines in mice. In addition, we investigated the underlying mechanisms by which ketamine inhibited necroptosis in vitro. We found that ketamine efficiently inhibited TNF-α-induced necroptosis by enhancing RIP1 ubiquitination and reducing the RIP1-RIP3 and RIP3-MLKL interactions, as well as the formation of necrosomes (Summary in Fig. 9). Our study suggests that ketamine can attenuate TNF-α-induced SIRS by inhibiting cecal damage and epithelial cell necroptosis. These findings may provide an experimental basis and represent an attractive therapeutic strategy for applying ketamine to treat inflammatory diseases including SIRS.

Epidemiologically, the burden of sepsis led to >11 million deaths annually and was announced as a global health priority by the World Health Assembly and WHO [34]. During sepsis and septic shock, aggravated systemic inflammation, especially SIRS, exerts a key effect on the life-threatening pathological conditions of dysfunctional organs [35]. Effector immune cells participate in the progression of SIRS and impair both innate and acquired immune responses to an infection *via* excessive synthesis and secretion of chemokines and proinflammatory cytokines [36]. Consistent with the fact that the upregulated production and release of cytokines and chemokines contribute to sepsis and organ dysfunction, such as TNF-α, IL-6, and IL-1β, are positively related to multiple organ dysfunction syndrome (MODS) and mortality [37–39]. In addition, the stress response induced by major procedures during the perioperative period could also damage the immune system by an excessive pro-inflammatory response [40]. Similarly, “cytokine storms” have also been reported in patients suffering from coronavirus disease 2019 (COVID-19) and once again has become a focus of public attention [41–43]. Collectively, this clinical evidence strongly suggests that novel therapeutic strategies targeting the restoration of immune system balance would contribute to reducing mortality in patients with sepsis.

Ketamine, a classic non-competitive inhibitor of the NMDAR, induces amnesia, anesthesia, and analgesia in medical practice [44]. Recently, the novel therapeutic effect of ketamine on depression and immunomodulation has been illustrated by a growing number of studies from both animal and clinical trials and has increased interest in its clinical application for the treatment of depression [45–48]. Considering that inhibiting inflammation is a component of the therapeutic mechanism of depression, explorations of the clinical benefits of ketamine in patients with inflammatory diseases, especially those with SIRS, are urgently needed.

Emerging findings from clinical and evidence-based medicine indicated that the use of ketamine perioperatively appeared to be more efficacious in larger procedures that led to extensive tissue damage and accelerated systemic inflammation and markedly inhibited the early postoperative IL-6 inflammatory response and natural killer (NK) cell activity [26, 49, 50]. Moreover, the immunoprotective effect of perioperative low-dose administration of ketamine [51, 52], as well as the alleviation of sepsis-associated MODS by inhibiting LPS-induced HGMB1 release, were confirmed by a previous publication [25]. The current research displayed that TNF-α could induce mouse death within 24 h, and interestingly, ketamine significantly promoted the survival of mice in a dose-dependent manner and alleviated the sickness response. Notably, the clinical benefits of ketamine to patients hospitalized for COVID-19 infection, which propels virus-induced immunological outbursts, have also been highlighted [53]. Clinical and laboratory outcomes encourage the preferential application of ketamine among the COVID-19 population over other sedative drugs because of its anti-inflammatory and immunomodulatory roles [54]. Collectively, ketamine seemingly is a novel candidate drug for immunomodulation in patients suffering from inflammatory pathophysiological processes, including SIRS and COVID-19.

The timing of ketamine treatment during SIRS still remained controversial. Previous studies indicated that ketamine has prophylactic and/or therapeutic effects against inflammatory responses induced by systemically administered LPS [55, 56], as well as organ protective effects in a variety of models of inflammatory diseases [57–61]. More recently, a study reported that combined prophylactic and therapeutic use of R-ketamine (10 mg/kg), as well as either prophylactic or therapeutic use of R-ketamine at a single dose of 15 mg/kg, did not reduce 14-day mortality after cecal ligation and puncture (CLP). However, combined prophylactic and therapeutic use of R-ketamine (15 mg/kg) significantly increased the 14-day survival rate [62]. Worth noting, our previous study suggested that ketamine presented neuroprotective effects as a potent inhibitor of necroptosis, providing a new theoretical and experimental basis for the application of ketamine in TNF-α-induced necroptosis-associated diseases [63]. In the current study, our findings indicated that the prophylactic ketamine at a single dose of 20 mg/kg exhibited the dominantly protective effect on survival rate in mice with acute SIRS-induced by TNF-α. These discrepancies might be derived from the process of SIRS (TNF-α induced acute SIRS *vs*. CLP-induced chronic SIRS), optical activity (S-ketamine *vs*. R-ketamine), as well as the dose of ketamine (20 mg/kg *vs*. 10 ~ 15 mg/kg). Pathophysiologically, gastrointestinal damage and cell death play roles as initiators or accelerators of SIRS. The previous study has confirmed that TNF-α caused severe oxidative stress and activation of caspase pathway, resulting in serious apoptosis and necrosis of the intestinal and cecal cells. In addition, the physical barrier function of the gastrointestinal mucosa can be influenced by great HMGB1 released by necrotic cells [64]. Follow-up innovational research revealed that cecal resection strikingly promoted TNF-α-induced lethality in mice by halting necroptosis [13]. Necroptosis, a lytic, proinflammatory cell death pathway described in many dysregulated human diseases, is characterized by activation of the receptor-interacting serine/threonine protein kinases RIP1 and RIP3 and MLKL [65]. Previous researches revealed that cecal damage in TNF-α-induced mice was dependent on RIP3-mediated necroptosis [13]. In addition, our previous research offered experimental evidence that TNF‐α both in vivo and in vitro may induce necroptosis in hippocampal neurons, followed by attenuation by ketamine *via* the control of ROS generation and MLKL phosphorylation [66]. In the present study, we determined that the anti-necroptotic mechanism of ketamine, which indirectly influences the kinase activity of RIP1 or its interaction with downstream proteins such as RIP3, was different from that of necrostatin-1, a classic inhibitor of necroptosis, by using the TNFR1, RIP1, RIP3, and MLKL dimer/poly systems. Moreover, the HT-29 cell line, an intestinal cell line, used to delineate the potential mechanism by which ketamine could be inhibiting necroptosis, as TNF treatment induces necroptosis in Nec-1 inhibitable fashion [67]. In the present study, we found ZVAD/LBW242/TNF-α administration enhances HT-29 cell death, which as stated in the previous study [67, 68]. In contrast, ketamine can provide protection for HT-29 against ZVAD/LBW242/TNF-α administration. Meanwhile, our study showed that the protection of ketamine was due to inhibiting the expression of p-MLKL. These outcomes indicate that Ketamine plays a potential protective role in the necroptosis of the intestinal cells. Together, our results suggested that ketamine improved the ubiquitination of RIP1 after TNF-α stimulation, and with further molecular understanding of the mechanisms, ketamine may be a promising candidate therapeutic strategy for SIRS-related organ dysregulation.

It has been reported that necroptosis takes part in some pathological processes including inflammation, atherosclerosis, and amyotrophic lateral sclerosis (ALS). Cecal damage was observed in TNF-α-treated mice, and this damage was dependent on RIP3-mediated necroptosis. Although necrostatin-1 has been shown to be an efficient inhibitor of necroptosis, no clinical drug has been developed for TNF-α-induced SIRS. This research investigated the protective roles of ketamine during TNF-α-induced SIRS. Unlike necrostatin-1, ketamine did not directly influence the kinase activity of RIP1 or its interaction with downstream proteins, such as RIP3. Ketamine improved the ubiquitination of RIP1 after TNF-α stimulation, but its mechanism is still unknown.

In summary, our research uncovered the interesting role of ketamine in TNF-α-induced SIRS, which inhibits necroptosis by affecting RIP1 ubiquitination levels and the formation of necrosomes and reducing the release of inflammatory cytokines. Our information provides new insight into the effect of ketamine on controlling the disease progression of SIRS. Our study may provide a theoretical and experimental basis for treating diseases characterized by SIRS-associated inflammatory factor storms and provide ideas for the clinical application of ketamine, with certain theoretical and clinical significance.

## Materials and methods

### Reagents and antibodies

Abcam provided rabbit anti-MLKL (ab184718), rabbit anti-phospho-RIP3 (T231, S232) (ab222320), rabbit anti-phospho-MLKL (S345)(ab196436), rabbit anti-TNFR1 (ab68160), rabbit anti-TRADD (ab110644), and rabbit anti-CIAP2 (ab32059) antibodies. Rabbit anti-RIP1 (3493 s) and rabbit anti-IκBα (9242) antibodies were purchased from Cell Signaling Technology. The mouse anti-GAPDH (AC033) antibody was purchased from AbClonal. Sigma provided the rabbit anti-RIP3 (PRS2283) antibody. Santa Cruz Biotechnology offered mouse anti-β-actin (sc-47778) antibody. Calbiochem (San Diego, CA, USA) offered Benzyloxycarbonyl-Val-Ala-Asp-fluoromethylketone (ZVAD; 627610). Sigma-Aldrich offered propidium iodide (PI; P4170). Murine TNF-α (PMC3015) was purchased from Thermo Fisher. Ketamine (1707031) was obtained from Gutian Pharmaceutical Co. Ltd.

### Animals

The housing of male C57BL/6 mice (18–25 g; Shanghai SLAC Laboratory Animal Co, Ltd.) was made in a given pathogen-free environment with a 12-h light/dark cycle with adequate food and water *ad libitum* at the Xiamen University Laboratory Animal Center. The Institutional Animal Care and Use Committee approved all mouse experiments according to good animal practice as defined by the Xiamen University Laboratory Animal Center (Approval No. XMULAC20190054). The binding of all investigators to the randomization assignment was made.

### SIRS mouse model

The random assignment of mice to the following groups was made. Mice in the TNF-α group were intravenously injected with 15 µg of TNF-α (75 µg/ml) diluted in endotoxin-free phosphate-buffered saline (PBS). Mice in the control group were injected with PBS alone. In the TNF-α + KET pretreatment group, ketamine was diluted in endotoxin-free PBS and intravenously injected at a dose of 20 mg/kg 20 min before TNF-α injection. Under constant observation, the animals were checked every 30 min. The mice were sacrificed at the indicated time, and the binding of investigator was made when the mice were injected with TNF-α and when the deaths were counted.

### Body temperature measurement

Previous studies have demonstrated that ear temperatures obtained using a convenient and noninvasive infrared thermometer are reliable, and the readings are similar and consistent with rectal temperatures [69, 70]. Moreover, this method should be safer and less stressful to the animals than standard rectal temperature measurements. In the present study, body temperature was measured by using a noncontact handheld transponder reader (Braun, Kronberg, Germany) and an implanted transponder. Briefly, the placement of mice was made on the laboratory technician’s palm with the base of the tail gently fixed during body temperature acquisition. The head of the handheld reader was held and inserted 2 ~ 3 cm into the animal’s ear, and slow circular movements were made until the temperature reading was displayed. Body temperature was read at 9 am every day during the experiment. Stress and discomfort were reduced by minimizing handling.

### Sickness behavior score

The sickness behavior score (SBS) uses physiological variables to evaluate the severity and mortality of sepsis. The hourly evaluation of animals is made for six indexes of sickness behavior: temperature change, water/sucrose preference, liquid intake, food intake, body weight, and movement. For each parameter, a score of 0 indicated healthy behavior, and a score of 1 indicated sickness behavior. Totally, the individual hourly SBS could differ from zero to six [71].

### Open field test

The mice were placed at the center of a cube-shaped arena (50 cm (L) × 50 cm (W) × 40 cm (H)), which was manually divided into an average of 16 square areas (3.125 cm (L) × 3.125 cm (W)) in a dimly illuminated testing room with indirect white lighting. The free locomotion of the mice was automatically monitored and recorded by a Noldus EthoVision XT (Noldus Information Technology, Wageningen, the Netherlands) for 5 min. The mean velocity was used as a parameter of locomotion, and the number of crossings of the areas (defined as entering the area with four paws) was a parameter to evaluate animal sickness by offline analysis [72]. The habituation of all animals to the testing room was made for 30 min before the start of the test. Between each test, the arena was cleaned by using a 75% ethanol spray to eliminate any residual odors.

### Histology and immunofluorescent staining

The mice were euthanized with isoflurane for 12 h after tail vein injection of TNF-α, and then the cecum was collected and fixed in 4% paraformaldehyde in PBS for 24 h. After dehydration with ethanol, cleared with xylene, the fixed tissues were embedded in paraffin blocks. The sections were stained with hematoxylin and eosin (H&E). The extent of cecal damage was evaluated by a previously illustrated method [73]. Identical settings were adopted to capture and possess representative images with a Leica Aperio Versa 200 at Xiamen University.

For immunofluorescent staining, the cecal tissue was removed and post-fixed for 2 ~ 4 h after perfusion, followed by 48-h cryoprotection at 4 °C in 0.1 M PB with 30% (w/v) sucrose. Transverse frozen spinal sections (30 μm thick) were cut in a cryostat (CM1800; Leica, Heidelberg, Germany), followed by serial collection after being embedded in mounting medium (OCT, Tissue-Tek, Sakura, Torrance, CA, USA). The rinsing of sections was made in 0.01 M phosphate-buffered saline (PBS, pH 7.2 ~ 7.4) three times (10 min each), followed by 30 min blocking with 5% fetal bovine serum (FBS) in 0.01 M PBS with 0.3% (v/v) Triton X-100 at room temperature. The 4-h incubation of sections was performed at RT, followed by overnight incubation at 4 °C, thereafter, with rabbit anti-p-MLKL antibody (1:100; Abcam, Cambridge, MA, USA) or rabbit anti-p-RIP3 antibody (1:100; Abcam). After three washes with 0.01 M PBS (10 min each), the 1-hour incubation of sections was made at RT with Alexa 488 or 594 donkey anti-ribbit IgG (1:1000; Abcam). Finally, the sections were mounted with mounting medium containing DAPI (Abcam) after washing three times with 0.01 M PBS (10 min each), and then obtained with a confocal laser scan microscope (Zeiss LSM 880 Airyscan; Carl Zeiss Microscopy GmbH, Promenade, Jena, Germany; 1 μm thick optical section).

### Determination of cytokines levels

HMGB1 (NBP2-62767, Novus), IFN-γ (CME0003, 4 A Biotech), IL-6 (CME0006, 4 A Biotech), and CXCL10 (CME0016, 4 A Biotech) concentrations in mouse serum were decided with enzyme-linked immunosorbent assay (ELISA, 4 A BIOTECH, China) kits according to the manufacturer’s instructions. The optical density of each sample was measured on an ELISA plate scanner (Multiskan Sky, Thermo Scientific, USA) at 450 nm [74].

### Western blot analysis of p-RIP3 and p-MLKL expression in vitro and in vivo

Western blot analysis was used to determine p-MLKL and GAPDH levels in the cecal tissue of mice in each group at 12 h after being injected with 15 μg of TNF-α or determine p-RIP3, p-MLKL, and GAPDH levels in L929 cells treated with nothing (Normal group), TNF-α + ZVAD, or TNF-α + ZVAD + ketamine for 3 h. Then, the cecum or L929 cells were collected and lysed for western blot analysis. To be brief, after separation by adopting 10% SDS‐PAGE gels with a marker (26616; Thermo Fisher Scientific, Inc.), proteins were transferred onto PVDF membranes (cat. no., ab133411; Abcam). 5% skim milk was adopted to block the membranes for 40 min to decrease nonspecific binding, followed by overnight incubation with primary antibodies (dilution, 1:1,000) at 4 °C. After 2-h incubation with horseradish peroxidase-conjugated mouse (cat. no., S0100; Beijing Lablead Biotech Co., Ltd.) or rabbit (cat. no., S0101; Beijing Lablead Biotech Co., Ltd.) secondary antibodies (dilution, 1:1,000) at room temperature, an improved chemiluminescence detection kit (cat. no., E1060; Beijing Lablead Biotech Co., Ltd.) was employed to visualize the protein bands. The band intensities were decided with ImageJ software (National Institutes of Health), which represented the expression of every protein.

### Immunoprecipitation and western blotting

3 × Flag-TNF-α (100 ng/ml) with or without ketamine (500 µg/ml) was employed to treat L929 cells for the given time. Then, the treatment of Flag-RIP1-expressing *rip1*-knockout (KO) L929 cells or Flag-RIP3-expressing RIP3-KO L929 cells was made with TNF-α (10 ng/ml) or TNF-α (10 ng/ml) plus ketamine (500 µg/ml) for the given time. After the cells were treated according to the experimental design, the 60-min lysis of cells was made with lysis buffer (20 mM Tris-HCl, pH 7.5, 150 mM NaCl, 1 mM Na_2_EDTA, 1 mM EGTA, 1% Triton X-100, 2.5 mM sodium pyrophosphate, 1 mM β-glycerophosphate, and 1 mM Na_3_VO_4_) on ice. The 30-min centrifugal of cell lysates was made at 20,000 *g*. Antibody-coupled beads were employed to immunoprecipitate the supernatant at 4 °C overnight. After immunoprecipitation, the beads were washed three times in lysis buffer, and after subsequent elution with SDS sample buffer, the immunoprecipitated proteins were boiled for 10 min at 100 °C and explored by western blotting as previously described [75].

### Cell culture

Colon cancer (HT-29) cell lines were cultivated in McCoy’s 5 A medium. The media was added with 10% FCS, 2 mM glutamine,100 units/ml penicillin, and 100 μg/ml streptomycin. The given amounts of recombinant human TNF (rhTNF) were added to induce Necroptosis. The 1-h treatment of HT-29 cells was made with 10 µM Smac mimetic LBW242 and 20 µM ZVAD before 24-h stimulation with 20 ng/ml rhTNF combining with various concentrations of ketamine. The CellTiter-Glo Luminescent Cell Viability Assay kit (Promega Corporation) was employed to determine cell survival on basis of the manufacturer’s guidance. Applying an ELISA kit to measure lactate dehydrogenase (LDH) (SEB864Mu, Cloud-Clone Corp) on basis of the manufacturer’s guidance. The Omega POLAR Star (BMG Labtech GmbH) was employed to record luminescence. Also, the collection and centrifugal of HT-29 cells were performed in 0.01 M PBS solution, and the removal of supernatant was realized. The dissolution of pellets was achieved in PRO-PREP solution by vortexing and ultrasonication for western blot analysis to test the level of MLKL phosphorylation.

Moreover, mouse fibroblast L929 cells were obtained from the ATCC. RIP1-KO L929 cells, RIP3-KO L929 cells, NEMO-KO L929 cells, and K376R RIP1 mutation cells were described previously [76, 77]. All cells were cultured in Dulbecco′s modified Eagle′s medium (Thermo Fisher Scientific) added with 10% fetal bovine serum (Gibco), 1% MEM nonessential amino acid solution (HyClone), and 100 U/ml penicillin/streptomycin at 37 °C in a humidified incubator with 5% CO_2_.

### Cell death assay

Cell death analysis was performed through the measurement of plasma membrane integrity and adding PI. After being added directly to the medium, PI (5 μg/ml) was incubated for 10 min to determine spontaneous cell death. The total number of PI-positive cells was counted under an inverted fluorescence microscope. Four fields of cells for every sample were counted. The information is shown as the percent of PI-positive cells per total cells counted. Cell death was also analyzed using an ELISA kit to measure lactate dehydrogenase (LDH) (SEB864Mu, Cloud-Clone Corp) or a CellTiter-Glo luminescent cell viability assay kit (G7571, Promega) according to the manufacturer’s protocols. Luminescent recording was performed with a POLAR star Omega (BMG Labtech, Durham, NC, USA).

### Reactive oxygen species measurement

A fluorometric intracellular ROS kit (cat. no., MAK143; Sigma-Aldrich; Merck KGaA) was employed to measure ROS levels on basis of the manufacturer’s guidance. A fluorescence microscope was adopted to view stained cells. The 3-hour treatment of cells was made with TNF-α + ZVAD or ketamine+TNF-α + ZVAD, and a CytoFLEXS flow cytometer (Beckman Coulter, Inc.) was used to measure fluorescence.

### TNFR1, RIP1, RIP3, and MLKL dimer/poly system

TNFR1, RIP1, RIP3, and MLKL fusion protein gene sequences with inducible dimerization/polymerization joints were constructed as previously described [78]. To produce fusion proteins, HBD*(G521R), *tTNFR1*, *RIP1ΔDD*, *RIP3-RHIM*^*mut*^, and *MLKLΔPD* were amplified by standard PCR from the related templates. The addition of an inducer resulted in dimerization/polymerization of the fusion proteins in eukaryotic cells, leading to necroptosis. This method can specifically induce dimerization/polymerization interactions among TNFR1, RIP1, RIP3, and MLKL through the addition of small molecule polymerization inducers.

### Statistical analysis

Prism software, version 7.0 (GraphPad Software Inc., San Diego, CA, USA) was adopted to analyze the information. The data are shown as the mean ± SEM. One-way ANOVA was employed to compare groups followed by Bonferroni’s or Dunnett’s posttest. The log-rank test was adopted to analyze survival curves, and *p*-values < 0.05 were regarded as significant.

## Supplementary information

Supplemental Figures

Original WB scan

## Data Availability

The authors declare that all data supporting the findings of this study are available within the article and from the corresponding author upon reasonable request.
