# Supplementary material for: Ketamine inhibits TNF-α-induced cecal damage by enhancing RIP1 ubiquitination to attenuate lethal SIRS
Source: Cell Death Discov. 2022 Feb 19;8:72. doi: 10.1038/s41420-022-00869-x (PMC8857635; doi:10.1038/s41420-022-00869-x)
Supplement: Supplementary file 1 — Supplemental Figures [file 41420_2022_869_MOESM1_ESM.pdf]

SFig. 1

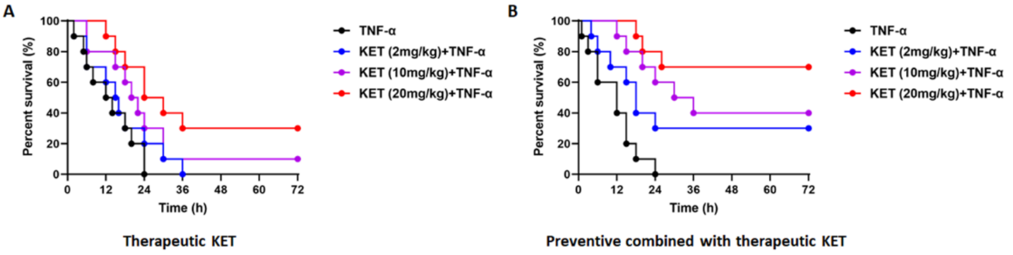

SFig. 1 (A) Effects of different therapeutic ketamine concentrations on the survival of model animals stimulated with TNF-α. (B) Effects of different preventive combined with therapeutic ketamine concentrations on the survival of model animals stimulated with TNF-α. n = 10/group.

SFig. 2

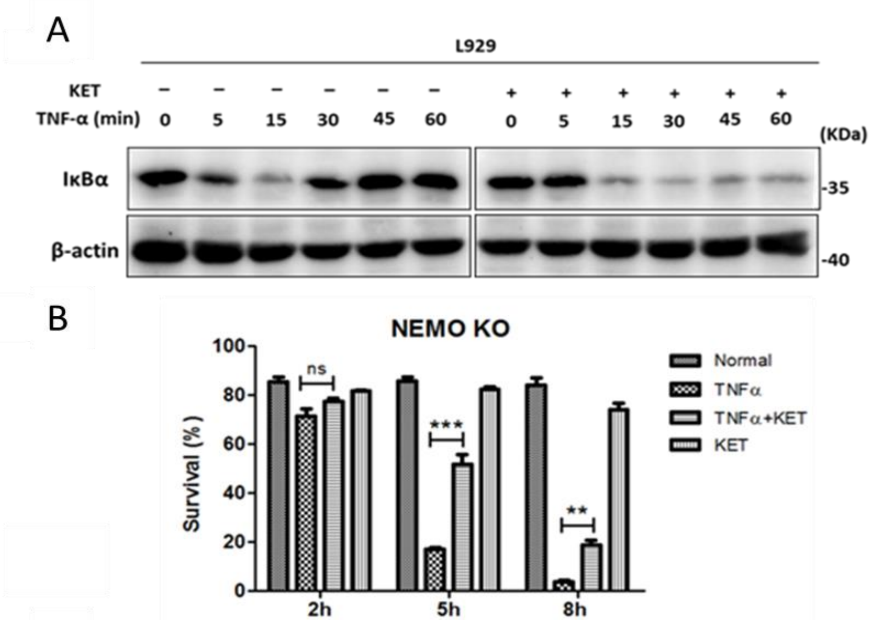

SFig. 2 (A) L929 cells were treated with TNF-α or with TNF-α plus ketamine for the indicated period of time and analyzed by western-blot with anti-IκB antibody. (B) NEMO KO L929 cells were treated with nothing, TNF-α, TNF-α plus ketamine or ketamine for the indicated period of time. Cells were stained with PI and cell survivals were analyzed by flow cytometry. The data is mean values of n=3 independent experiments and the error bars are mean ± s.e.m., ***P*<0.01, ****P*<0.001, ns: no significant difference.

SFig. 3

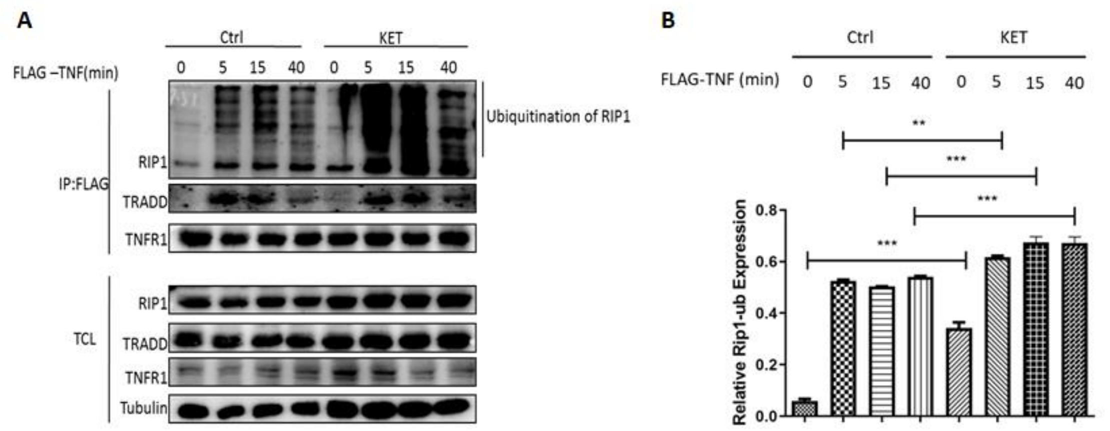

SFig. 3 (A) KET (20 mg/kg) was pre-treated in SIRS mice-induced by 3×Flag-TNF-α, and then were sacrificed at 0, 5, 15, and 40 min. Cecal tissue lysates were immunoprecipitated with mouse anti-Flag M2 beads and analyzed by western blotting with Flag-RIP1. (B) Statistic analysis for relative ubiquitination level of Rip1 in cecal tissue with or without KET pre-treatment in SIRS mice-induced by 3×Flag-TNF-α. All data are shown as the mean ± SEM. n = 3/group. ** $P<0.01$, *** $P<0.001$.
