# Supplementary material for: Ketamine inhibits TNF-α-induced cecal damage by enhancing RIP1 ubiquitination to attenuate lethal SIRS
Source: Cell Death Discov. 2022 Feb 19;8:72. doi: 10.1038/s41420-022-00869-x (PMC8857635; doi:10.1038/s41420-022-00869-x)

Fig 3

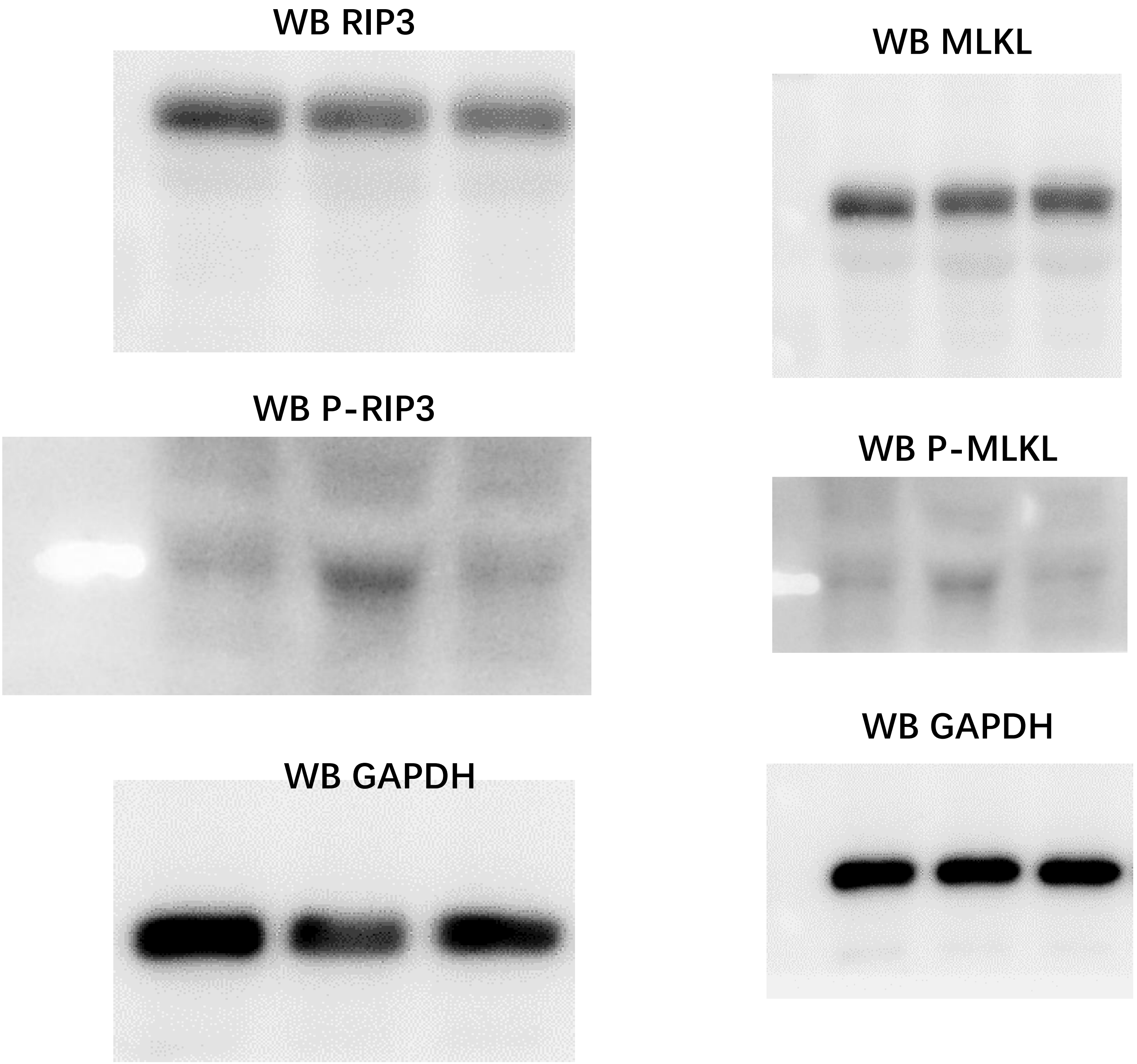

Fig 4

WB P-MLKL

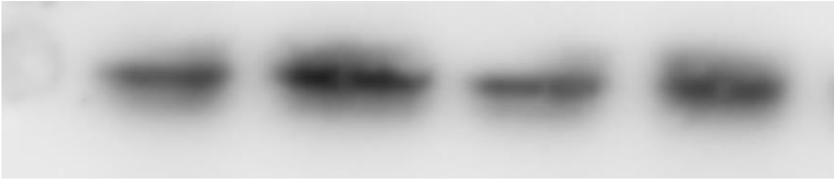

WB GAPDH

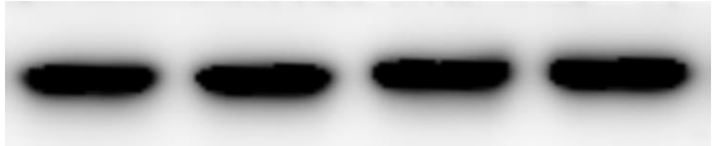

Fig 6

C

WB P-RIP3

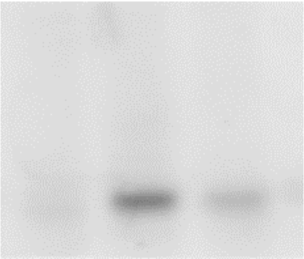

WB GAPDH

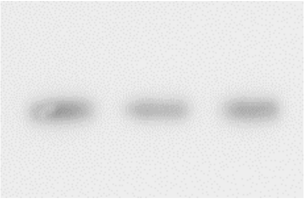

D

WB P-MLKL

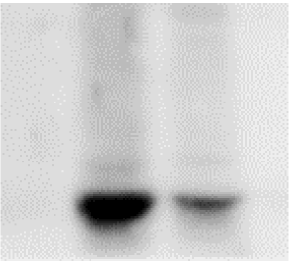

WB GAPDH

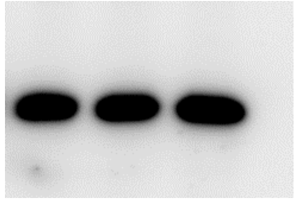

Fig 8 A

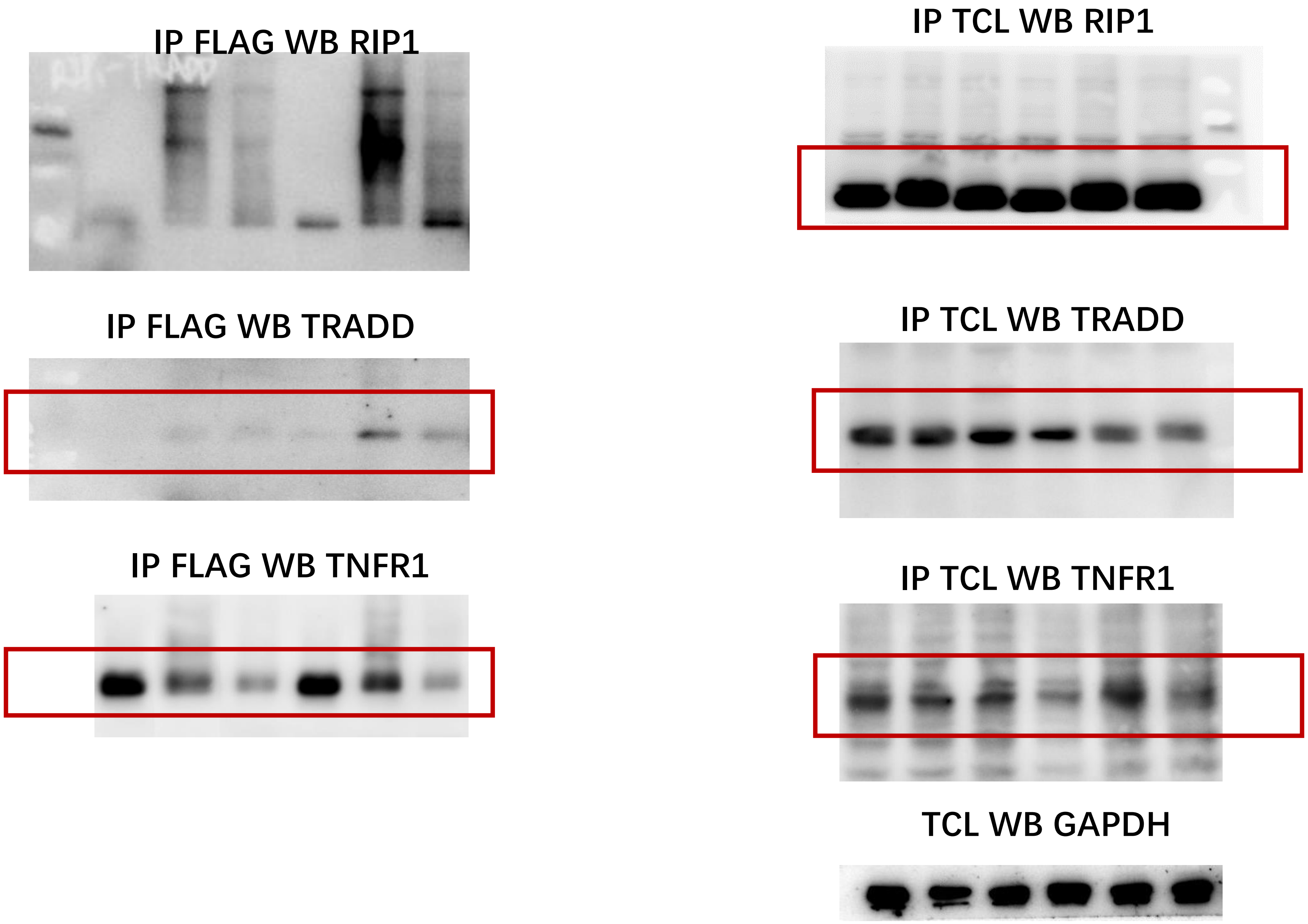

IP FLAG WB RIP1
IP FLAG WB TRADD
IP FLAG WB TNFR1
IP TCL WB RIP1
IP TCL WB TRADD
IP TCL WB TNFR1
TCL WB GAPDH

Fig 8 **B**

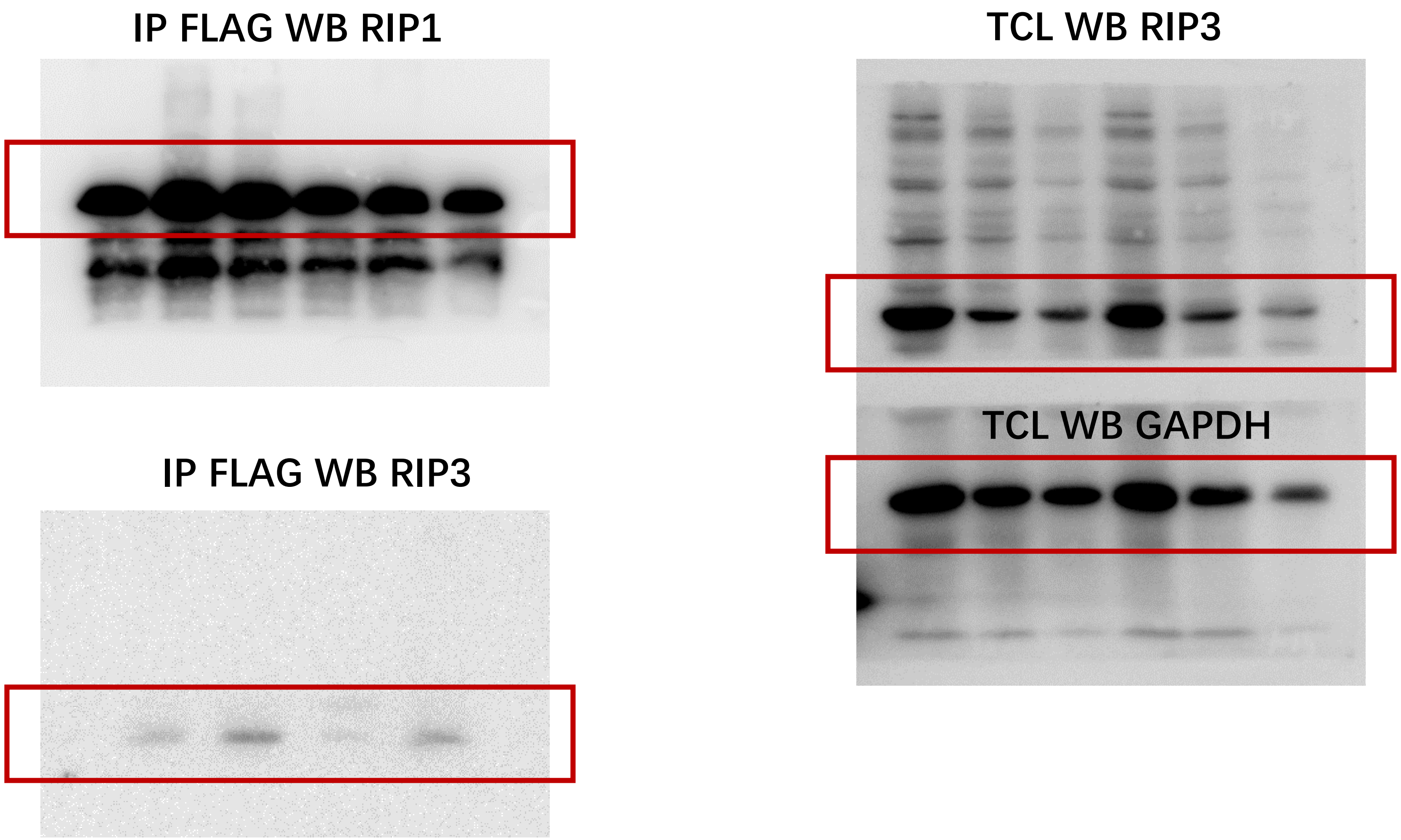

Fig 8 C

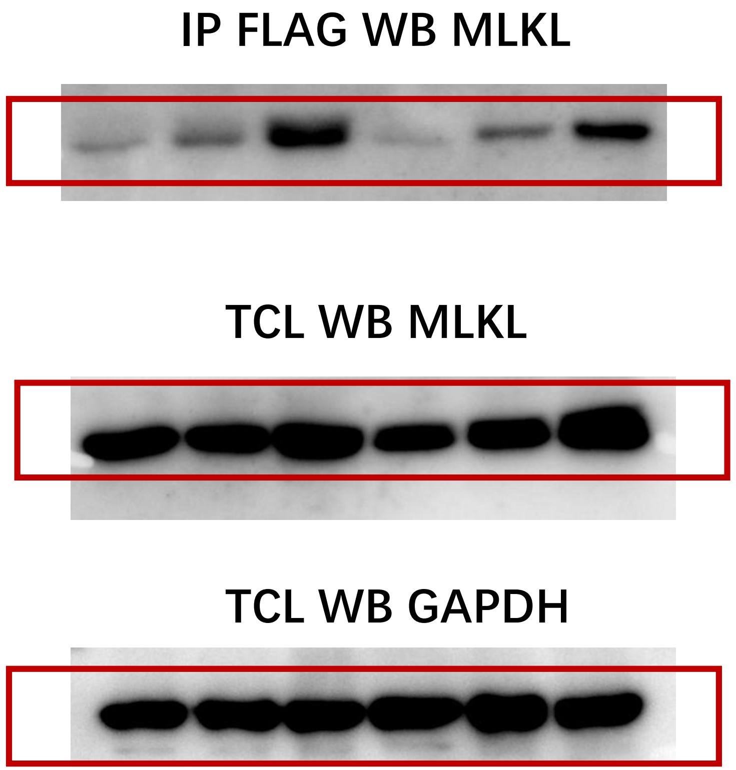

Fig S1 A/B

WB IκBα

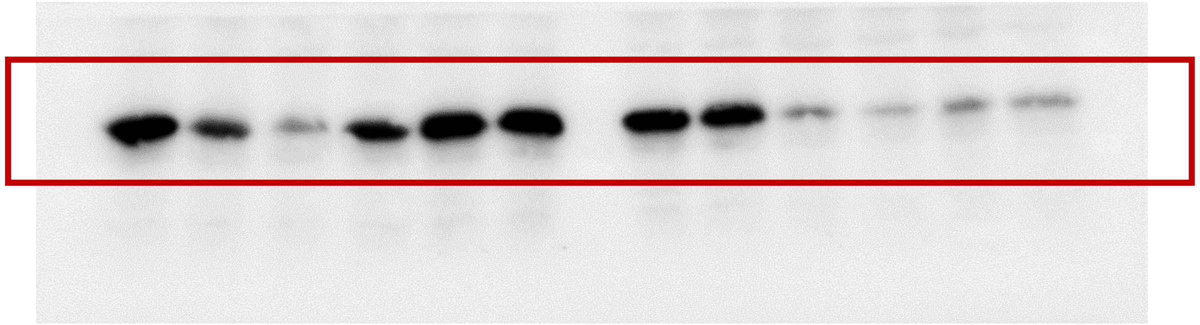

WB β-actin

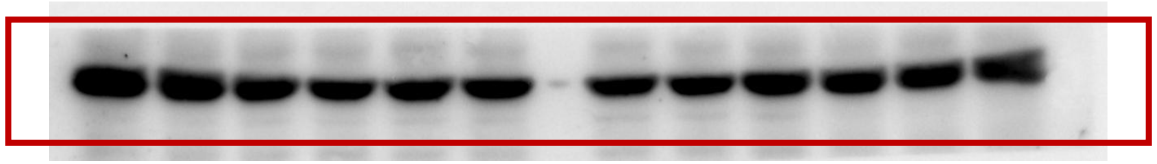

Supplement: Supplementary file 2 — Original WB scan [file 41420_2022_869_MOESM2_ESM.pdf]
